# Fully Prepared

**Published:** 1862-10

**Authors:** 


### FULLY PREPARED.

Who? and how? Why, J. M. Brown, of the original Dental Depot, corner of 4th and Walnut sts. Dr. B. has just returned from the East with a full supply of dental materials. His long experience in the dental furnishing line enables him to select just what is needed by the profession, and that of the very best quality. This is the oldest establishment of the kind in the West, and consequently has at its head a more extended experience. All orders for goods will be promptly filled. T.

